# Effect of Chromium Nanoparticles and Switching from a High-Fat to a Low-Fat Diet on the Cecal Microenvironment in Obese Rats

**DOI:** 10.3390/nu15143118

**Published:** 2023-07-12

**Authors:** Bartosz Fotschki, Katarzyna Ognik, Ewelina Cholewińska, Katarzyna Grzelak-Błaszczyk, Kamil Myszczyński, Magdalena Krauze, Jerzy Juśkiewicz

**Affiliations:** 1 Division of Food Science, Institute of Animal Reproduction and Food Research, Polish Academy of Sciences, Tuwima 10, 10-748 Olsztyn, Poland; b.fotschki@pan.olsztyn.pl; 2 Department of Biochemistry and Toxicology, Faculty of Animal Sciences and Bioeconomy, University of Life Sciences in Lublin, Akademicka 13, 20-950 Lublin, Poland; katarzyna.ognik@up.lublin.pl (K.O.); ewelina.cholewinska@up.lublin.pl (E.C.); magdalena.krauze@up.lublin.pl (M.K.); 3 Institute of Food Technology and Analysis, Lodz University of Technology, Stefanowskiego 2/22, 90-537 Lodz, Poland; katarzyna.grzelak-blaszczyk@p.lodz.pl; 4 Molecular Biology Laboratory, Institute of Animal Reproduction and Food Research, Polish Academy of Sciences, Tuwima 10, 10-748 Olsztyn, Poland; k.myszczynski@pan.olsztyn.pl

**Keywords:** gut microbiota profile, microbiota activity, chromium nanoparticles, secondary bile acids, obesity, rat

## Abstract

Previous studies showed that chromium nanoparticles (Cr-NPs) might be used as dietary compounds against some obesity-related disorders; however, there is little information on how these compounds influence the gut microenvironment. The aim of this study was to investigate whether the negative effects of a high-fat diet in the large intestine of rats might be mitigated by switching to a low-fat diet and supplementation with Cr-NPs. Microbiota sequencing analysis revealed that the main action of the Cr-NPs was focused on changing the gut microbiota’s activity. Supplementation with nanoparticles decreased the activity of β-glucuronidase and enzymes responsible for the hydrolysis of dietary oligosaccharides and, thus, lowered the concentration of short-chain fatty acids in the cecum. In this group, there was also an elevated level of cecal lithocholic acid. The most favorable effect on the regulation of obesity-related disorders was observed when a high-fat diet was switched to a low-fat diet. This dietary change enhanced the production of short-chain fatty acids, reduced the level of secondary bile acids, and increased the microbial taxonomic richness, microbial differences, and microbial enzymatic activity in the cecum. To conclude, supplementation of a high-fat diet with Cr-NPs primarily had an effect on intestinal microbial activity, but switching to a low-fat diet had a powerful, all-encompassing effect on the gut that improved both microbial activity and composition.

## 1. Introduction

Unhealthy eating habits leading to obesity, as a continuing challenge to public health efforts, are major risk factors for cardiovascular morbidity and mortality [1]. Obesity is not only associated with an array of chronic civilization-related diseases but also affects the homeostasis and proper balance of intestinal microbial communities [2]. Although micronutrients are required in minute quantities in the diet, they play an important role in improving host health status, including the large intestinal environment [3,4]. It should be stressed that chromium has gained popularity as a nutritional supplement for obese consumers who enjoy good and fatty food and whose low self-motivation for switching to a less energy-dense diet results in poor weight loss and reduced health-promoting effects. Currently, the most popular form of chromium used in dietary supplements is picolinate, an organic compound composed of trivalent Cr [5]. The available literature indicates that its bioavailability, even though it is not high, is greater than that of its inorganic counterparts, such as CrCl_3_ [6]. Our understanding of the interaction between the gut microbiota and host health has recently improved dramatically. It is well known that the amount and type of dietary fat significantly affect the gut microbiome [2], and a high-fat diet causes detrimental outcomes on microbial diversity and activity [7]. In our previous study [8], a high-fat diet caused a thwarting effect on the short-chain fatty acid content in the rat cecum, alongside an increase in the cecal digesta pH and an enhanced activity shift toward enzymes characteristic of less-beneficial bacteria (β-glucuronidase, β-glucosidase). It is well known that treatment with a high-fat diet substantially enhances intestinal bile acid flow [9]. Some primary bile acids are converted by the microbial community to their secondary derivatives, which have important physiological properties; moreover, some bile acids possess strong antimicrobial activity [10]. Those processes may exert selective additional influence to change the gut microbiota enzymatic activity, the production of short-chain fatty acids (SCFAs), and the microbial composition. Yokota and coworkers [11] confirmed the role of bile acids in the control of the gut microbiota in vivo and underlined the importance of bile acid as a factor interacting with the gut microbiota and, thus, affecting host pathophysiology.

The effects of exposure to some metals on the gut microbiota remain poorly characterized. As this microbiota creates a critical interface between the external environment and the host’s cells, it may play an important role in host outcomes during exposure. Richardson et al. [12] observed only modest changes in the rat microbiota composition in response to high doses of chromium (sodium dichromate) vs. huge changes noted upon arsenic, cadmium, and nickel exposures. It is well known that the intestinal microbiota shows an imbalanced status in patients with obesity, nonalcoholic fatty liver disease, and type 2 diabetes [13,14]. Guo et al. [15] showed that organic chromium derived from the chelation of *G. frondosa* polysaccharide-chromium (III) exhibited excellent hypoglycemic and hypolipidemic activities in diabetic mice induced by high-fat diet feeding and streptozotocin (STZ) injection by regulating liver-related gene transcription and modulating the intestinal microbiota. Previous studies on Wistar rats even showed that the gut bacteria provided the first line of defense to the body by converting toxic Cr(VI) to less toxic Cr(III), and the latter may act similarly to a prebiotic [16]. Those authors revealed the development of large intestine antibiotic resistance upon Cr(III), and the effects were most marked with *Pseudomonas* sp. and least with *E. coli*. Antibiotic resistance also developed with *Lactobacillus* sp. [16]. Yang et al. [17] reported beneficial effects when providing supplementary Cr-enriched *Bacillus subtilis* to mice, manifested as enhanced regulation of body growth, increased tissue organic Cr concentrations, beneficially altered cecal microbiota, and enhanced insulin receptor expression to produce significant changes in plasma biochemistry. The recent work of Dworzański and coauthors [18] noted, in addition to the negative aspects of a high-fat dietary regimen, some positive effects of different types of chromium supplementation on microbial metabolism. Interestingly, among the chromium forms used in that study, the Cr nanoparticles most efficiently diminished the cecal/fecal activity of the potentially detrimental bacterial enzymes β-glucosidase and β-glucuronidase.

The development of nanotechnology has ushered in new trends in many fields. The term ‘nanoparticle’ refers to a material with a particle size of less than 100 nm. It is believed that the lack of electric charge and the small size of chromium nanoparticles allows their properties to be modified in living organisms and their bioavailability to be improved relative to their standard counterparts. Recently, nanoparticles have emerged as important players in modern science, including the nutritional status of the host in health and disease. In the last decade, numerous nutrition experiments have also been carried out using metal nanoparticles, showing that the biological response of the organism depended on the size of the particles, the method by which they were produced, the dosage applied, and the length of administration [19]. Li et al. [20] reported high nanoparticle chromium absorbability in pigs fed diets containing chromium chloride, chromium picolinate, nanoparticle chromium chloride, and nanoparticle chromium picolinate. Our recent work showed that both dietary high-fat treatment and the nanoparticle chromium form (vs. Cr picolinate) significantly enhanced fecal Cr excretion, thus, diminishing Cr digestibility and retention in Wistar rats [21]. There are two sides to dietary nano-Cr, one beneficial and one detrimental, that have been reported by our own research group as well as several authors conducting in vivo experiments [22,23,24,25]. Some questions have been answered, but such research needs further well-designed examination. As far as the animal models are concerned, there is considerable similarity between the rodent and human internal organs and microbiota functioning, in the latter case at the division (super kingdom) level. Although in vivo data on metal nanoparticles obtained from rats may not be entirely transferable to humans, it must be stressed that such new information advances our knowledge [26].

In the present study, the main intention was to provide new insight with regard to the question of whether negative large intestinal effects associated with chronic consumption of a high-fat diet could be mitigated by switching to a low-fat diet accompanied by dietary chromium addition. Taking into account an urgent need to establish the true action of dietary nanoparticles in the gastrointestinal tract, paramount attention was given to chromium nanoparticles, and their action was compared to that of the commonly used dietary supplement chromium picolinate. The present study assessed the negative or positive impact of dietary habit change as well as supplemental Cr on the microbiota living in the lower gut of obese hosts.

## 2. Materials and Methods

Chromium nanoparticles (Cr-NPs; Sky Spring Nanomaterials Inc., Houston, TX, USA) were characterized as follows: 99.9% nanoparticles, 70 nm (mean size), 7 m^2^/g (mean surface area), and 8.9 g/cm^3^ (true density). The purity of the chromium picolinate (Cr-Pic; Sigma, Poznań, Poland) was 98%.

**Figure 1:**
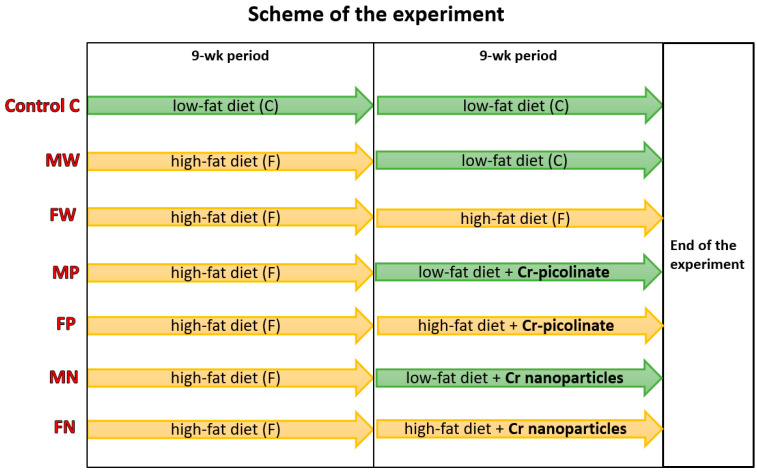
Scheme of the nutritional experiment.

Male outbred Wistar rats (Cmdb:Wi CMDB) were used in the present study (n = 84). The in vivo experimental schema was approved by the National Ethics Committee for Animal Experiments (Approval No. 73/2021). Rats were kept individually in cages with constant comfortable temperature (22 ± 1 °C), relative humidity 60 ± 5%, a 12 h day/night period, and a ventilation rate of 15 air changes per hour. The study lasted 18 weeks and consisted of two 9-week periods, namely an initial and experimental period. Two types of diet were applied to rats: a low-fat diet (C diet; 15%, 22%, and 63% of kcal from dietary protein, fat, and carbohydrates, respectively) and a high-fat diet (F diet; 11%, 49%, and 40% of kcal from dietary protein, fat, and carbohydrates, respectively). Moreover, two forms of dietary additional chromium were used: standard Cr-picolinate and novel Cr nanoparticles (Cr-Pic and Cr-NPs, respectively) at the pharmacologically relevant dose of 0.3 mg/kg body weight. The details of the experimental dietary protocol were described in recently published work aimed at hepatic metabolism [22]. In brief, the rats during the experimental period were divided into 7 groups (n = 12 each): Groups C and MW were fed the low-fat C diet without Cr addition; Group FW was fed the high-fat F diet without Cr addition; Groups MP and MN were fed the C diet with additional Cr-Pic and Cr-NPs, respectively; Groups FP and FN were fed the F diet with Cr-Pic and Cr-NPs, respectively (Table 1). The diets were formulated according to the American Institute of Nutrition [27]. During the initial 9 weeks of feeding, chromium supplementation was not applied, and the C group was subjected to a standard low-fat diet, while the remaining rats (Groups M, MP, FP, MN, and FN) were fed a high-fat F diet to induce obesity. The scheme of the experiment is shown in Figure 1.

After the 18-week feeding, the animals were deprived of feed for 12 h and then anesthetized with an i.p. mixture of ketamine and xylazine (K, 100 mg/kg BW; X, 10 mg/kg BW) in physiological saline. The rats were euthanized by cervical dislocation. After laparotomy, the cecum was dissected and weighed as the tissue and digesta masses.

Samples of cecal contents were used for immediate analysis (ammonia, dry matter, primary and secondary bile acids, and short-chain fatty acids (SCFAs)), while the rest of the cecal digesta was transferred to tubes, frozen in liquid nitrogen, and then stored at −70 °C. In fresh cecal digesta, ammonia was extracted, trapped in a solution of boric acid in Conway’s dishes, and determined by direct titration with sulfuric acid. The dry matter of the digesta was determined at 105 °C. The concentrations of SCFAs in cecal digesta samples were determined by gas chromatography (Shimadzu GC-2010, Kyoto, Japan). The samples (0.2 g) were mixed with 0.2 mL of formic acid, diluted with deionized water, and centrifuged at 7211× *g* for 10 min. The supernatant was loaded onto a capillary column (SGE BP21, 30 m × 0.53 mm) using an on-column injector. The initial oven temperature was 85 °C, which was raised to 180 °C at 8 °C/min and held for 3 min. The temperatures of the flame ionization detector and the injector were 180 °C and 85 °C, respectively. The sample volume for GC analysis was 1 μL. The concentrations of cecal putrefactive SCFAs (PSCFAs) were calculated as the sum of iso-butyric acid, iso-valeric acid and valeric acid. All SCFA analyses were performed in duplicate. Pure acetic, propionic, butyric, iso-butyric, iso-valeric, and valeric acids were obtained from Sigma Co. (Poznan, Poland), and their mixture was used to create a standard plot and then to calculate the number of individual acids. This additional set of pure acids was included in each GC run of samples at five-sample intervals to maintain calibration.

The bile acids (BAs) in the cecal digesta were assessed using a Shimadzu LC system (Kyoto, Japan) coupled to a Shimadzu LCMS-2020 mass spectrometer (Kyoto, Japan). Chromatographic separation was conducted using an Avantor ACE, C18-amide, 75 × 2.1 mm, 2.7-µm column by gradient elution with 0.01% (*v*/*v*) formic acid in water (solvent A), and a mixture of methanol and acetonitrile at a proportion of 1 to 9 (*v*/*v*) (solvent B). The column temperature was set at 45 °C, the flow rate was 0.35 mL/min, and the gradient program was as follows: 0–8 min, 40% (*v*/*v*) B; 8–14 min, 50% (*v*/*v*) B; 14–15.3 min, 50–100% (*v*/*v*) B; 15.3–17.4 min, 40% (*v*/*v*). The injection volume was 1 µL. Mass data acquisitions were performed using LabSolutions 5.109 software (Shimadzu). Quantitative analysis was carried out with external standards (0.01–1 μM) that had been used to construct linear calibration curves with correlation coefficients of 0.994–0.997. All analyses were performed in triplicate for each sample.

Bacterial enzyme activity was measured by the rate of p- or o-nitrophenol release from their nitrophenyl glucosides in the cecal digesta (α- and β-glucosidase, α- and β-galactosidase, β-glucuronidase, cellobiosidase, α-arabinopyranidase, and α-arabinofuranosidase). The following substrates were used: p-nitrophenyl-α-D-glucopyranoside (for α-glucosidase), p-nitrophenyl-β-D-glucopyranoside (for β-glucosidase), α-nitrophenyl-α-D-galactopyranoside (for α-galactosidase), o-nitrophenyl-β-D-galactopyranoside (for β-galactosidase), p-nitrophenyl-β-D-glucuronide (for β-glucuronidase), and p-nitrophenyl-β-D-xylopyranoside (for β-xylosidase). The reaction mixture contained 0.3 mL of a substrate solution (5 mM) and 0.2 mL of a 1:10 (*v*/*v*) dilution of the cecal sample in 100 mM phosphate buffer (pH 7.0) after centrifugation at 7211× *g* for 15 min. Incubation was carried out at 37 °C, and p-nitrophenol was quantified at 400 nm and at 420 nm (o-nitrophenol concentration) after the addition of 2.5 mL of 0.25 M cold sodium carbonate. The enzymatic activity was expressed as μmol of product formed per hour per gram of digesta. The abovementioned procedure concerns the activities of bacterial enzymes released from bacterial cells into the cecal environment. The total enzymatic activity of selected enzymes, which includes the intracellular and extracellular enzyme activities, was also assessed. Therefore, another cecal digesta sample was mechanically disrupted by vortexing with glass beads (212–300 μm in diameter; four periods of 1 min with 1 min cooling intervals on ice) using the FastPrep^®^-24 homogenizer (MP Biomedicals, Santa Ana, CA, USA). The resulting mixture was centrifuged at 7211× *g* for 15 min at 4 °C. The supernatant was used for the enzyme assay described above. Intracellular enzyme activity was calculated by comparing total enzyme activity with the activities of bacterial enzymes secreted into the intestinal environment. To prepare the calculation formulas, the model curves for PNP and ONP (standard solution in 100 mM phosphate buffer pH 7.0, 40 mg/L) were used, and appropriate equations were obtained. All analyses were performed in duplicate.

The diversity of the cecal rat microbiota was evaluated using next-generation sequencing of the 16S rRNA bacteria-specific variable region according to a previously described method [28]. Samples of cecal digesta were collected in an Eppendorf tube and stored at −80 °C prior to DNA extraction and sequencing. Total DNA was extracted from the samples using the QIAamp Fast DNA Stool Mini Kit (Qiagen, Hilden, Germany) following the manufacturer’s instructions. DNA was used to assess the microbiota profile by amplification of the V3–V4 region of the 16S rRNA gene using primers and protocols according to the 16S metagenomic sequencing library preparation instructions. The paired-end sequencing reaction (2 × 250 bp) was performed using the Illumina MiSeq platform according to the manufacturer’s instructions.

### Statistics

Two-way ANOVA and Student’s t test were used to analyze the results. The t test was applied to compare a single experimental group (MW, FW, MP, FP, MN, and FN) with the control C group. Two-way ANOVA was applied to assess the effects of the main factors and the interaction between them (groups MW, FW, MP, FP, MN, and FN). When ANOVA indicated significant treatment effects, means were evaluated using Duncan’s multiple range test. Data were checked for normality before statistical analysis was performed. Differences with *p* ≤ 0.05 were considered to be significant (Statistica 12.0; StatSoft Corp., Krakow, Poland).

Regarding the results of cecal bacterial sequencing, statistical analyses were performed according to a protocol from a previous study [28]. Briefly, alpha diversity measures (Shannon index) were calculated using the phyloseq [29] R package, and differences in the alpha diversity measures were analyzed using ANOVA with the post hoc Tukey test within the stats [30] R package. Multidimensional scaling (MDS) of the Bray–Curtis dissimilarity distance matrix was performed using phyloseq [29]. To test the beta diversity (Bray–Curtis) differences among groups of samples, permutational multivariate analysis of variance (PERMANOVA) followed by pairwise PERMANOVA was calculated within the vegan [31] and pairwise Adonis [32] R packages. Differential ASV abundance analyses based on negative binomial distribution were performed using the DESeq2 [33] R package. P values were adjusted for multiple testing by false discovery rate using the Benjamini–Hochberg method [34].

## 3. Results

The control C rats had significantly lower starting BW as they were fed the control low-fat diet (*p* < 0.05 vs. all experimental groups; *t*-test). Two-way ANOVA showed that rats fed the F diet had a higher final BW than that in the M treatment. The lowest fat tissue percentage (measured by nuclear magnetic resonance analysis) was noted in the MW and MP groups (*p* < 0.05 vs. FW, FP, and FN animals). The Cr × D interaction with regard to the lean tissue percentage showed that the FN group did not differ significantly vs. the MN and MP groups. The highest value for lean tissue percentage was attributed to the MW rats (*p* < 0.05 vs. F, FP, and FN groups). The abovementioned data were not provided in the present work in the tables due to their presentation in the recent study by Fotschki et al. [22]. A description of the results on animal growth parameters is presented in Supplemental File S1.

The two-way ANOVA showed that, regardless of Cr addition, the relative cecal tissue and digesta weights were diminished and the ammonia concentration and digesta pH values were enhanced in rats fed the high-fat diet in comparison to the M treatment (animals fed the low-fat diet) (Table 2). The C rats had higher relative cecal tissue weight than all three groups fed the high-fat diet (FW, FP, and FN; *t*-test). The cecal ammonia concentration was higher in all groups that in the C rats, except groups MW and MN. In comparison to the control C rats, the FW animals had a higher cecal pH value. The two-way ANOVA revealed that irrespective of additional Cr, the high-fat dietary treatment caused a significant decrease in the cecal concentrations of acetic, propionic, and butyric acid and total SCFAs compared to those in the M treatment (Table 3). The propionic acid profile (expressed as a percentage of the sum of SCFAs) was higher in the F treatment (*p* < 0.05 vs. M treatment; the opposite was true in the case of butyric acid). Regardless of the diet type, the addition of Cr-NPs caused a significant decrease in cecal acetic, propionic, butyric, iso-valeric, and total SCFA concentrations (*p* < 0.05). The *t* test analysis showed that the C group was higher than all the experimental groups with respect to the acetic and butyric acid and total SCFA concentrations in the cecal digesta (in the case of C_4_, except for the MP group). The acetic acid profile was reduced in the MP and FP groups vs. the C group. The FW, FP, and MN groups were characterized by enhanced propionic acid profile percentages (*p* < 0.05 vs. C). The butyric acid profile was diminished in the FW, FP, MN, and FN groups compared to the control C animals (*p* < 0.05).

Irrespective of addition of Cr, the high-fat dietary treatment (treatment F) substantially increased the cecal concentrations of β-muricholic acid (β-MCA), ω-muricholic acid (ω-MCA), cholic acid (CA), deoxycholic acid (DCA), and total analyzed bile acids (*p* < 0.05 vs. M treatment; Table 4). The F treatment significantly diminished the cecal concentration of ursodeoxycholic acid (UDCA) and caused a tendency toward lowered levels of hyodeoxycholic acid (HDCA) (*p* < 0.05 and *p* = 0.052, respectively, vs. M treatment). The dietary addition of Cr-NPs caused a tendency toward diminished cecal HDCA, regardless of the diet type (*p* = 0.091). A significant Cr × D interaction for the cecal lithocholic acid (LCA) level showed that the FW group was significantly higher than the MW group. The *t*-test revealed that only the FW and FN groups had higher ω-MCA cecal concentrations vs. the C group. The FW group had a decreased UDCA content, the FN group had diminished HDCA levels, and the FP group had an enhanced DCA cecal concentration in comparison to those of the C group (*p* < 0.05).

The extracellular activity of bacterial α-glucosidase in the cecal digesta was enhanced, while the intracellular activity was decreased with the F diet, irrespective of Cr addition (*p* < 0.05 vs. M treatment; Table 5). The calculated α-glucosidase release rate was enhanced with the F treatment (*p* < 0.05 vs. the M group). When Cr addition regardless of diet type was considered, Cr-NP addition resulted in decreased extracellular and total activities and release rate percentages of bacterial cecal α-glucosidase. Compared to the C group (*t*-test), the FW group had higher activity and the MN group had lower extracellular activity of α-glucosidase. The extracellular and total activities as well as the release rate of bacterial β-glucosidase were decreased in the groups fed the high-fat diet, irrespective of the addition of Cr. Two-way ANOVA showed that regardless of the diet type, dietary chromium nanoparticles caused a significant decrease in the extracellular activity and release rate of bacterial β-glucosidase in the cecal digesta (*p* < 0.05 vs. treatments without Cr and with Cr-Pic). The C group exceeded the FW, FP, MN, and FN groups with respect to the extracellular and total activities of bacterial β-glucosidase (*p* < 0.05; *t*-test). A decrease in the extracellular and total activity of bacterial cecal α-galactosidase was evident with the F treatment, irrespective of Cr supplementation (*p* < 0.05 vs. M; Table 6). When dietary Cr was considered, the extracellular activity and release rate of that enzyme were enhanced in the Cr-NPs treatment vs. the W and P groups (without Cr and with Cr-Pic, respectively). The α-galactosidase extracellular and total activities were decreased in all groups vs. C (*t*-test), except the MW and MP groups. The α-galactosidase release rate was decreased in groups MW, MN, and FN vs. the C group (*p* < 0.05; *t*-test). Two-way ANOVA showed that regardless of Cr supplementation, an increase in the extracellular and total activities of bacterial cecal β-galactosidase was associated with the F treatment compared to the M (*p* < 0.05). Irrespective of diet type, Cr-NP supplementation caused a decrease in β-galactosidase extracellular and total activities as well as the release rate percentage (*p* < 0.05; vs. the W and P treatments). The t test revealed that the FW and FP groups were higher than the C group with respect to the extracellular and total activities of β-galactosidase. The release rate percentage was enhanced only in the FW animals (*p* < 0.05 vs. C; *t*-test).

Dietary treatment with Cr-NPs caused a significant decrease in the extracellular activity and release rate of bacterial cecal β-glucuronidase compared to the treatment with no additional Cr and with Cr-Pic, irrespective of diet type (Table 7). Compared to the C group animals (*t*-test), the FW and FP rats had higher extracellular activity, and the FW, FP, and MN animals had higher total activity of bacterial cecal β-glucuronidase (*p* < 0.05). A Cr × D interaction showed that the MW group was higher than the remaining groups with respect to β-xylosidase extracellular activity in the cecal digesta (*p* < 0.05). Regardless of the addition of Cr, the rats fed the high-fat diet had lower total activity of bacterial β-xylosidase (*p* < 0.05 vs. rats on the low-fat diet treatment). The t test revealed that all experimental groups, except MW, had lower extracellular activity in terms of bacterial cecal β-xylosidase than the C group.

**Figure 2:**
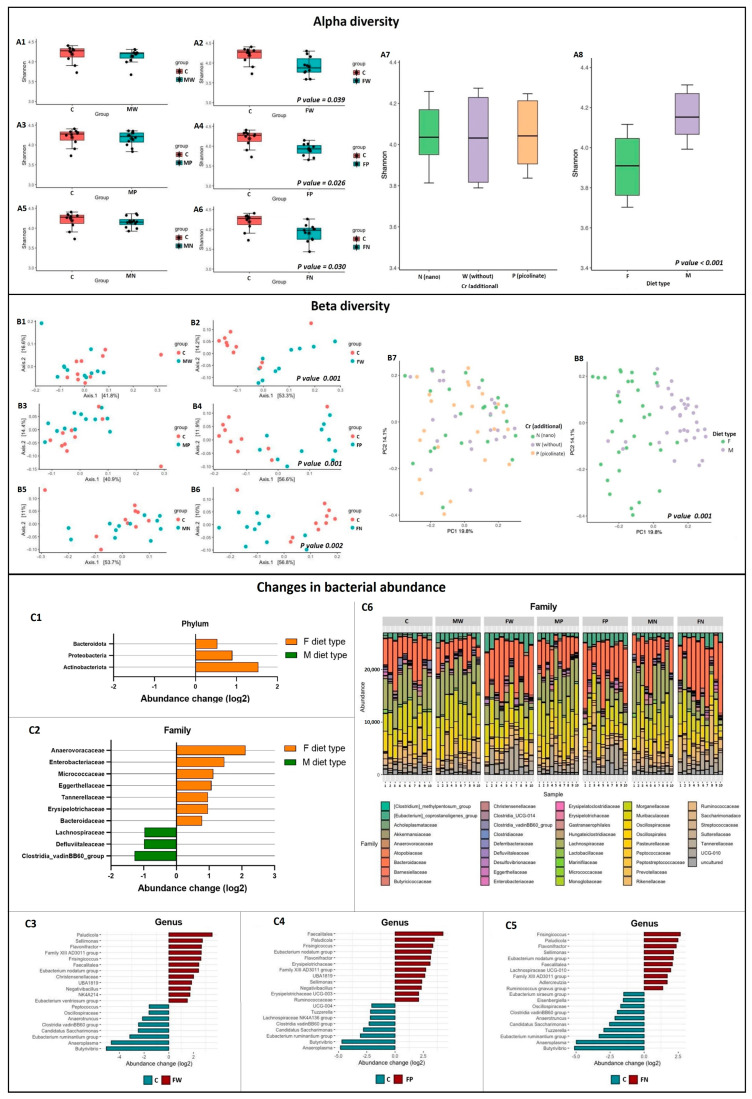
NGS analyses of cecal microbiota diversity (n = 10 per group). Details about the feeding period and how the experiment was performed were described in the Materials and Methods section. M, type of diet that includes a change from a high-fat diet to a low-fat diet; F, type of diet that includes staying on a high-fat diet for the entire duration of the experiment; C, rats fed a standard diet for laboratory rodents; W, rats fed a diet without supplementation with chromium; P, rats fed a diet with chromium picolinate; N rats fed a diet with chromium nanoparticles. The alpha diversity calculated with the Shannon index for all groups (**A1**–**A6**) in comparison with Control C, addition of chromium (**A7**) and diet type (**A8**). The horizontal line inside the graphs represents the median, while the asterisk represents the mean. Individual dots indicate Shannon values for individual samples. The beta diversity calculated with PCoA based on Bray–Curtis distances for all groups (**B1**–**B6**) in comparison with Control C, addition of chromium (**B7**) and diet type (**B8**). The graphs for changes in bacterial abundance show significant variations at the phylum (**C1**), family (**C2**), and genus (**C3**–**C5**) levels. The comparisons within groups with significant differences (*p <* 0.05) as indicated by analyses of beta diversity. The graph (**C6**) illustrates changes at bacterial family levels in individual samples.

Next-generation sequencing (NGS) analyses showed that supplementation with the examined types of Cr had no effect on the cecal microbiota profile (Figure 2). The main dietary factor responsible for changes in the microbiota taxonomic composition was related to switching away from a high-fat diet. The alpha diversity analysis clearly indicates a lower number of bacterial taxa estimated by the Shannon index in the FP, FW, and FN rats (*p* < 0.05 vs. C rats). Additionally, comparison between dietary treatment F (high-fat diet) and M (switching away from the high-fat diet) showed that the high-fat diet considerably decreased microbial taxonomic richness (*p* < 0.001; Figure 2, alpha diversity). The analysis of differences in terms of microbiome composition (beta diversity) showed similar effects. The PCoA based on Bray–Curtis distances demonstrated significant changes in the bacterial community for all groups with the F dietary treatment in comparison to the C group as well as the M dietary treatment (Figure 2, beta diversity). The FW, FP, and FN groups had 39, 55, and 32 significantly different bacterial taxa, respectively, at the genus level in comparison with the C group. There were no significant differences in bacterial taxa at the genus level between rats from the Group C and Group M dietary treatments. The top 20 most variable taxa from each comparison were selected to present changes in bacterial abundance at the genus level. All animals fed a high-fat diet for 18 weeks (F diet) had six predominant bacterial genera with considerably elevated abundances in comparison to those of the C group, i.e., *Paludicola*, *Sellimonas*, *Flavonifractor*, *Frisingicoccus*, *Faecalitalea*, and *Eubacterium nodatum* groups. In comparison to the control diet, there were also specific changes in the bacterial genera in each group with the F dietary treatment. In rats from the FP group, the abundance changes in *Ruminococcaceae* and *Erysipelotrichaceae* UCG-003 were considerably elevated. In the FN group, considerably higher abundance changes were observed for *Ruminococcus gnavus* group, *Adlercreutzia*, and *Lachnospiraceae* UCG-010. However, in the group without the addition of Cr (FW group), the abundance changes in *Eubacterium ventriosum* group and *Christensenellaceae* were significantly elevated. Moreover, the beta diversity analyses at the genus level demonstrated that except for *Adlercreutzia*, all considered differences were related to bacterial genera from the Firmicutes phylum. However, the analyses at the phylum level in the group with the F dietary treatment showed considerably higher values of abundance changes only for *Actinobacteriota*, *Bacteroidota*, and *Proteobacteria* (*p* < 0.05 vs. M dietary treatment, Figure 2, Changes in bacterial abundance). At the bacterial family level, the F dietary treatment increased the abundances of *Anaerovoracaceae*, *Micrococcaceae*, *Tannerellaceae*, *Eggerthellaceae*, *Bacteroidaceae*, *Enterobacteriaceae*, and *Erysipelotrichaceae* (*p* < 0.05 vs. M dietary treatment).

## 4. Discussion

The role of the gut microbiota is crucial in many metabolic diseases, including those linked to obesity [7]. These microorganisms are involved in the production of SCFAs that take part in the regulation of many mechanisms associated with the development of obesity [3,4]. By fermenting indigestible polysaccharides, intestinal anaerobic bacteria generate SCFAs, such as acetate, propionate, and butyrate, as primary end products. These bacterial products are important factors involved in the prevention and treatment of metabolic disorders related to disturbances in energy metabolism [7]. A previous study on rats showed that an obesogenic diet reduced the concentration of SCFAs in the cecum and, thus, elevated the digesta pH [8]. This study showed that switching from a high-fat diet to a standard diet enhanced the content of SCFAs, decreased the digesta pH, and reduced the level of ammonia in the cecum. Elevated concentrations of ammonia and pH in the gastrointestinal tract may promote disturbances in the functioning of intestinal cells and the growth of pathogens that colonize neutral or slightly alkaline environments [35,36]. Moreover, alterations in the cecal ammonia levels may be related to greater dietary protein usage and undigested protein compounds. NGS analyses supported the observed changes in the cecum of rats. The abundance changes in *Proteobacteria* and *Bacteroidota* at the phylum level as well as at the genus level of bacteria belonging to *Firmicutes* were considerably elevated in the groups with the F dietary treatment (18-week high-fat diet). These results are in agreement with other studies reporting that taxa from *Bacteroides* and *Proteobacteria* exhibited reduced growth in lower pH environments and that taxa from Firmicutes were more tolerant [37]. Furthermore, switching away from a high-fat diet increased the cecal tissue mass to the level observed in the control group. This effect might be associated with an increased production of SCFAs, which are energy sources for epithelial cells [38].

Another dietary factor that may modulate the activity and profile of the gut microbiota is supplementation with Cr [12,18]. In this study, among the examined forms of Cr, only supplementation with Cr-NPs had a considerable effect on intestinal microbiota activity. The nanoparticles, regardless of dietary treatment, reduced the concentration of SCFAs, i.e., acetic, propionic, and butyric acid, in the cecum, and these changes were not related to variation in the intestinal bacterial composition. Regardless of the Cr forms used in the study, there were no significant differences in the analyses of alpha- and beta diversity, which represent microbial taxonomic richness and differences. Only deeper analyses at the bacterial genus level showed a significant effect of dietary supplementation with Cr in the F dietary treatment when compared to the control diet. It has been noted that in rats fed diets with all Cr forms and F dietary treatment, the change in the *Butyrivibrio* abundance was considerably lower in comparison to that in the control diet. This gut bacterium is one of the main butyrate-producing strains [39]. Changes in the intestinal microbial profile partially explain the observed inhibitory effect of Cr and a high-fat diet on the SCFA production in the cecum. Nevertheless, it should be noted that the effect on the microbial composition is mostly focused on the impact of a high-fat diet. The alterations in microbiota activity caused by Cr might be associated with an increased reactive surface area related to the smaller size of the nanoparticles [19] and interaction with bacteria in the gastrointestinal tract. Taking into account the lack of significant changes in the NGS analysis, it might be assumed that the main effect of Cr-NPs on the intestinal microbiota was focused on their activity. A similar effect on intestinal microbial activity was observed in another nutritional study on rats fed a diet with high doses of chromium (sodium dichromate) [12].

Other factors that might have a strong impact on intestinal microbiota activity are bile acids. The main role of these acids is to act as emulsifiers to facilitate the absorption of lipids from the diet [11]. There are two main groups of bile acids: primary bile acids synthesized from cholesterol in the liver and secondary bile acids produced by the intestinal microbiota. These acids might affect bacterial membranes, denature proteins, chelate iron and calcium, and damage DNA, thus, exerting bacteriostatic and bactericidal effects on the intestinal microbiota [2]. However, microorganisms possess specific defense mechanisms to counter the antimicrobial effects of bile acids. An et al. [2] analyzed bacterial susceptibility and showed that bile acids exhibited different inhibitory effects on the growth of intestinal bacteria. Bile acids, such as CA, CDCA, DCA, and LCA, may affect the growth of probiotic microbes, which are the main producers of SCFAs in the intestine environment [37]. In this study, the considerably high levels of bile acids induced by a high-fat diet were mostly regulated when the diet was switched away from the high-fat diet to a standard diet. However, when the high-fat diet was supplemented with Cr nanoparticles, the level of LCA was considerably elevated, whereas HDCA was reduced in the cecum. LCA is one of the most toxic bile acids that can contribute to the development of gut inflammation and colorectal cancer [40]. One of the intestinal mechanisms regulating the level of LCA is related to the ability of bacteria to hydroxylate C-6 and convert it to less toxic HDCA [41]. HDCA is resorbed from the intestine and transported to the liver, where, after glucuronidation, it might be eliminated along with the feces. Due to the observed changes in the bile acid profile, it might be assumed that the conversion of LCA to HDCA was interrupted when the high-fat diet was enriched with Cr-NPs. Furthermore, diets with nanoparticles considerably reduced the activity of bacterial β-glucuronidase in the cecum. This enzyme is able to convert xenobiotics, harmful substances, and conjugated bile acids back into hydrophobic and more toxic forms, causing undesired changes in the intestine [42]. Additionally, Dworzański et al. [18], in a nutritional study on rats, showed that a diet with Cr nanoparticles reduced the activity of bacterial β-glucuronidase. Analyses of the extracellular and intracellular activity of this enzyme revealed that the potential of the microbiota to produce β-glucuronidase was not changed; however, the mechanism of enzyme secretion into the intestinal microenvironment may be affected. The same effect was observed for the activity of α- and β-glucosidase and α- and β-galactosidase. These enzymes assist in the hydrolysis of indigestible oligosaccharides derived from the diet and, thus, indirectly affect the supply of substrate for the synthesis of bacterial SCFAs in the intestine [43]. Moreover, low galactosidase activity may increase colonic fermentation and gas production, which is undesirable, especially in people with irritable bowel syndrome [44]. Low bacterial enzymatic activity as well as unchanged bacterial enzymatic potential in the cecum are associated with no significant change in the gut microbial composition and, thus, partially explain the effect of Cr-NPs on lowering SCFA production.

The effect of switching from a high-fat diet to a standard diet on bacterial enzymatic activity in the cecum was mostly related to variation in the composition of bile acids, which are the main factors responsible for the changes in the intestinal microbial environment [45]. In the rats under the M dietary treatment, considerably lower concentrations of CA and DCA were found in the cecum. These bile acids had a significant inhibitory effect on the growth of potentially prebiotic bacterial strains, e.g., *Lactobacillus casei*, *Clostridium butyricum*, and *Bacillus mesentericus* [2], which are SCFA-producing strains and exhibit increased enzymatic activity for the hydrolysis of oligosaccharides [43]. Therefore, the reduction in DCA and CA through switching from a high-fat diet to a standard diet may partially explain the increased activity of β-glucosidase, α-galactosidase, and β-xylosidase in the cecum. However, this mechanism is not in line with the decreased activity of α-glucosidase and β-galactosidase in rats from the M dietary treatment group. It appears that the effect of pre-feeding a high-fat diet may exhibit more permanent changes in the microbiota profile and their enzymatic activity.

## 5. Conclusions

Switching to a low-fat diet accompanied by dietary Cr-NP addition may mitigate disorders induced by a high-fat diet in the large intestine environment. The analyses of the gut microbiota composition showed that the effect of the nanoparticles was limited to affecting the enzymatic activity of the microbiota. The favorable effect of nanoparticles was associated with a decrease in the extracellular activity of β-glucuronidase. Nevertheless, the most effective action in the regulation of gut-related disorders induced by a high-fat diet was shown by switching to a low-fat diet. This change in diet favorably increased the production of SCFAs, reduced the level of undesired secondary bile acids (DCA and LCA), favorably increased microbial taxonomic richness and differences, and, thus, increased microbial enzymatic activity responsible for the hydrolysis of oligosaccharides. To summarize, supplementation with chromium nanoparticles mainly affected intestinal microbial activity, whereas changing eating habits to a low-fat diet showed strong wide-ranging intestinal action, including an improvement in microbial activity and composition. Nevertheless, our previous study showed that chromium nanoparticles together with a switch away from high-fat/low-fiber dietary habits enhances the pro-healthy regulation of liver lipid metabolism and inflammation [22]. Therefore, from a broader perspective of counteracting disorders induced by high-fat diet consumption, supplementation with Cr nanoparticles in conjunction with changing dietary habits may still be a reasonable approach to counteracting obesity-related disorders.

## Figures and Tables

**Table 1:** The composition of the experimental diets (g/100 g).

	C	F	MP	FP	MN	FN
Casein	14.8	14.8	14.8	14.8	14.8	14.8
DL-methionine	0.2	0.2	0.2	0.2	0.2	0.2
Cellulose	8	3	8	3	8	3
Choline chloride	0.2	0.2	0.2	0.2	0.2	0.2
Cholesterol	0.3	0.3	0.3	0.3	0.3	0.3
Vitamin mix ^1^	1	1	1	1	1	1
Mineral mix ^1^	3.5	3.5	3.5	3.5	3.5	3.5
Maize starch	64	52	64	52	64	52
Rapeseed oil	8	8	8 (with Cr-Pic)	8 (with Cr-Pic)	8 (with Cr-NP)	8 (with Cr-NP)
Lard	0	17	0	17	0	17

^1^ According to the requirements of the diet AIN-93G-VM [27].

**Table 2:** The cecal parameters in rats fed experimental diets (n = 12 per group) *.

	Cecal Tissue	Cecal Digesta	Cecal Ammonia	Cecal pH
	g/100 g BW	g/100 g BW	mg/g	
Control C	0.146	0.292	0.244	7.41
Two-way ANOVA:				
MW	0.146	0.285	0.280	7.41
FW	0.121 ^#^	0.230	0.341 ^#^	7.67 ^#^
MP	0.136	0.303	0.308 ^#^	7.45
FP	0.113 ^#^	0.210 ^#^	0.344 ^#^	7.47
MN	0.136	0.293	0.292	7.35
FN	0.125 ^#^	0.254	0.357 ^#^	7.57
SEM	0.003	0.012	0.008	0.022
Cr (additional)				
W (without)	0.133	0.257	0.310	7.53
P (picolinate)	0.124	0.256	0.326	7.46
N (nano)	0.130	0.273	0.324	7.46
*p* value	0.348	0.817	0.629	0.297
D (diet type)				
M	0.139 ^a^	0.293 ^a^	0.293 ^b^	7.40 ^b^
F	0.119 ^b^	0.231 ^b^	0.347 ^a^	7.57 ^a^
*p* value	<0.001	0.012	<0.001	<0.001
Interaction Cr × D				
*p* value	0.480	0.668	0.683	0.072

* Details about the feeding period and how the experiment was performed were described in the Materials and Methods section. M, type of diet that includes a change from a high-fat diet to a low-fat diet; F, type of diet that includes staying on a high-fat diet for the entire duration of the experiment; C, rats fed a standard diet for laboratory rodents; W, rats fed a diet without supplementation with chromium; P, rats fed a diet with chromium picolinate; N rats fed a diet with chromium nanoparticles. ^a,b^ The mean values within a row with different superscript letters were significantly different (*p* < 0.05, two-way ANOVA followed by Duncan’s multiple range test). ^#^ Data significantly different from the control C group (*p* < 0.05, *t*-test).

**Table 3:** Cecal concentration (µmol/g) and profile (% of total SCFAs) of short-chain fatty acids in rats (n = 12 per group) *.

	C2	C3	C4i	C4	C5i	C5	PSCFA	SCFA	C2 Profile	C3 Profile	C4 Profile
Control C	39.9	10.5	1.05	6.22	1.14	1.03	3.22	59.8	66.7	17.5	10.4
Two-way ANOVA:											
MW	33.2 ^#^	9.39	0.903	4.84 ^#^	1.14	0.955	3.00	50.4 ^#^	66.1	18.5	9.49
FW	27.2 ^#^	8.76 ^#^	1.00	3.28 ^#^	1.26	0.968	3.23	42.5 ^#^	64.2	20.5 ^#^	7.72 ^#^
MP	32.7 ^#^	9.74	1.06	5.25	1.22	1.08	3.36	51.0 ^#^	63.9 ^#^	19.1	10.3
FP	26.9 ^#^	9.12	1.00	3.29 ^#^	1.22	1.01	3.23	42.6 ^#^	63.1 ^#^	21.5 ^#^	7.71 ^#^
MN	28.2 ^#^	8.58 ^#^	0.926	3.81 ^#^	0.959	0.921	2.81	43.4 ^#^	65.1	19.7 ^#^	8.79 ^#^
FN	24.2 ^#^	7.30 ^#^	0.974	2.94 ^#^	1.10	0.903	2.97	37.6 ^#^	64.8	19.4	7.89 ^#^
SEM	0.695	0.193	0.026	0.160	0.030	0.027	0.072	0.994	0.362	0.268	0.195
Cr (additional)											
W (without)	30.2 ^a^	9.07 ^a^	0.953	4.06 ^a^	1.19 ^a^	0.961	3.11	46.4 ^a^	65.1	19.5	8.60
P (picolinate)	29.8 ^a^	9.42 ^a^	1.03	4.26 ^a^	1.22 ^a^	1.04	3.29	46.8 ^a^	63.5	20.3	9.00
N (nano)	26.3 ^b^	7.93 ^b^	0.950	3.37 ^b^	1.02 ^b^	0.911	2.88	40.5 ^b^	64.9	19.5	8.33
*p* value	<0.001	0.001	0.381	0.001	0.028	0.152	0.093	<0.001	0.204	0.420	0.259
D (diet type)											
M	31.3 ^a^	9.23 ^a^	0.963	4.63 ^a^	1.10	0.984	3.05	48.3 ^a^	65.0	19.1 ^b^	9.52 ^a^
F	26.1 ^b^	8.39 ^b^	0.993	3.17 ^b^	1.19	0.959	3.14	40.9 ^b^	64.0	20.4 ^a^	7.77 ^b^
*p* value	<0.001	0.013	0.584	<0.001	0.183	0.647	0.551	<0.001	0.211	0.014	<0.001
Interaction Cr × D											
*p* value	0.493	0.643	0.456	0.082	0.631	0.821	0.575	0.621	0.690	0.096	0.123

* Details about the feeding period and how the experiment was performed were described in the Materials and Methods section. M, type of diet that includes a change from a high-fat diet to a low-fat diet; F, type of diet that includes staying on a high-fat diet for the entire duration of the experiment; C, rats fed a standard diet for laboratory rodents; W, rats fed a diet without supplementation with chromium; P, rats fed a diet with chromium picolinate; N rats fed a diet with chromium nanoparticles. C2, acetic acid; C3, propionic acid; C4i, iso-butyric acid; C4, butyric acid; C5i, iso-valeric acid; C5, valeric acid; PSCFA, putrefactive SCFA (sum of C4i, C5i and C5); SCFA, total short-chain fatty acid. ^a,b^ The mean values within a row with different superscript letters were significantly different (*p* < 0.05, two-way ANOVA followed by Duncan’s multiple range test). ^#^ Data significantly different from the control C group (*p* < 0.05, *t*-test).

**Table 4:** Cecal bile acid concentration (mg/g digesta) of rats fed experimental diets (n = 12 per group) *.

	α-MCA	β-MCA	ω-MCA	CA	HDCA	CDCA	DCA	LCA	UDCA	Total BA
Control C	2.51	5.60	3.70	0.756	10.7	0.034	3.31	0.010	0.435	27.2
Two-way ANOVA:										
MW	2.29	4.54	3.88	0.638	11.1	0.017	2.96	0.009 ^c^	0.378	25.8
FW	2.69	7.09	8.17 ^#^	2.30	7.26	0.025	4.94	0.027 ^a b #^	0.317^#^	32.8
MP	1.97	6.02	4.85	1.07	9.03	0.017	3.04	0.019 ^b #^	0.418	26.4
FP	2.23	5.76	6.84	1.90	8.27	0.015	5.24 ^#^	0.019 ^b #^	0.320	30.6
MN	2.34	5.63	4.76	1.47	7.25	0.050	2.21	0.029 ^a b #^	0.413	24.2
FN	3.09	8.54	9.83 ^#^	2.51	6.08 ^#^	0.029	4.29	0.036 ^a #^	0.335	34.7
SEM	0.161	0.402	0.586	0.246	0.480	0.006	0.229	0.002	0.015	1.248
Cr (additional)										
W (without)	2.49	5.81	6.03	1.47	9.16	0.021	3.95	0.018	0.347	29.3
P (picolinate)	2.10	5.89	5.85	1.49	8.65	0.016	4.14	0.019	0.369	28.5
N (nano)	2.72	7.08	7.29	1.99	6.67	0.039	3.25	0.032	0.374	29.5
*p* value	0.392	0.409	0.609	0.687	0.091	0.347	0.223	<0.001	0.771	0.956
D (diet type)										
M	2.20	5.40 ^b^	4.50 ^b^	1.06 ^b^	9.11	0.028	2.74 ^b^	0.019	0.403 ^a^	25.5 ^b^
F	2.67	7.13 ^a^	8.28 ^a^	2.24 ^a^	7.20	0.023	4.82 ^a^	0.027	0.324 ^b^	32.7 ^a^
*p* value	0.205	0.049	0.004	0.040	0.052	0.728	<0.001	0.003	0.017	0.009
Interaction Cr × D										
*p* value	0.848	0.267	0.600	0.822	0.383	0.704	0.979	0.027	0.896	0.624

* Details about the feeding period and how the experiment was performed were described in the Materials and Methods section. M, type of diet that includes a change from a high-fat diet to a low-fat diet; F, type of diet that includes staying on a high-fat diet for the entire duration of the experiment; C, rats fed a standard diet for laboratory rodents; W, rats fed a diet without supplementation with chromium; P, rats fed a diet with chromium picolinate; N rats fed a diet with chromium nanoparticles. CA, cholic acid; DCA, deoxycholic acid; CDCA, chenodeoxycholic acid; LCA, lithocholic acid; α-, β-, and ω-MCA, α-, β-, and ω-muricholic acid, respectively; UDCA, ursodeoxycholic acid; HDCA, hyodeoxycholic acid. ^a,b,c^ Mean values within a row with different superscript letters were significantly different (*p* < 0.05, two-way ANOVA followed by Duncan’s multiple range test). ^#^ Data significantly different from the control C group (*p* < 0.05, *t*-test).

**Table 5:** The activity of bacterial α-glucosidase and β-glucosidase and their release rate in the cecum of rats fed experimental diets (n = 10 per group) *.

	α-Glucosidase				β-Glucosidase			
	Extracellular	Intracellular	Total	Release Rate	Extracellular	Intracellular	Total	Release Rate
Control C	16.6	8.32	24.9	67.2	13.9	3.44	17.3	80.9
Two-way ANOVA:								
MW	16.9	8.53	25.3	65.6	13.2	3.16	16.4	81.6
FW	21.0 ^#^	5.03 ^#^	26.1	80.3 ^#^	9.39 ^#^	3.00	12.4 ^#^	76.5
MP	17.0	7.89	24.8	68.1	13.0	2.44	15.5	84.4
FP	17.3	5.47 ^#^	22.8	75.8 ^#^	9.12 ^#^	3.11	12.2 ^#^	75.0
MN	13.4 ^#^	10.0	23.4	57.1 ^#^	10.9 ^#^	3.09	14.0 ^#^	78.2
FN	14.8	7.44	22.3	66.8	7.61 ^#^	3.28	10.9 ^#^	69.3 ^#^
SEM	0.444	0.313	0.409	1.234	0.366	0.179	0.413	1.138
Cr (additional)								
W (without)	18.8 ^a^	6.82 ^b^	25.7 ^a^	73.0 ^a^	11.3 ^a^	3.08	14.4	79.1 ^a^
P (picolinate)	17.1 ^a^	6.66 ^b^	23.8 ^a b^	71.9 ^a^	11.1 ^a^	2.77	13.8	79.7 ^a^
N (nano)	14.1 ^b^	8.73 ^a^	22.8 ^b^	61.9 ^b^	9.27 ^b^	3.18	12.4	73.8 ^b^
*p* value	<0.001	0.002	0.012	<0.001	0.003	0.616	0.060	0.045
D (diet type)								
M	15.7 ^b^	8.83 ^a^	24.5	63.6 ^b^	12.4 ^a^	2.90	15.3 ^a^	81.4 ^a^
F	17.7 ^a^	5.98 ^b^	23.7	74.3 ^a^	9.71 ^b^	3.13	11.8 ^b^	73.6 ^b^
*p* value	0.014	<0.001	0.302	<0.001	<0.001	0.512	<0.001	<0.001
Interaction Cr × D								
*p* value	0.126	0.588	0.323	0.373	0.889	0.631	0.850	0.652

* Details about the feeding period and how the experiment was performed were described in the Materials and Methods section. M, type of diet that includes a change from a high-fat diet to a low-fat diet; F, type of diet that includes staying on a high-fat diet for the entire duration of the experiment; C, rats fed a standard diet for laboratory rodents; W, rats fed a diet without supplementation with chromium; P, rats fed a diet with chromium picolinate; N rats fed a diet with chromium nanoparticles. ^a,b^ Mean values within a row with different superscript letters were significantly different (*p* < 0.05, two-way ANOVA followed by Duncan’s multiple range test). ^#^ Data significantly different from the control C group (*p* < 0.05, *t*-test).

**Table 6:** The activity of cecal bacterial α-galactosidase and β-galactosidase and their release rate in the cecum of rats (n = 10 per group) *.

	α-Galactosidase				β-Galactosidase			
	Extracellular	Intracellular	Total	Release Rate	Extracellular	Intracellular	Total	Release Rate
Control C	24.2	4.46	28.7	85.0	75.7	18.6	94.3	80.4
Two-way ANOVA:								
MW	22.2	5.82	28.1	79.7 ^#^	82.4	17.6	100	83.0
FW	19.7 ^#^	4.61	24.3 ^#^	81.8	94.3 ^#^	16.0	110 ^#^	85.8 ^#^
MP	22.1	4.77	26.9	82.3	81.9	16.8	98.6	83.1
FP	18.3 ^#^	2.66	21.0 ^#^	87.2	95.7 ^#^	17.7	113 ^#^	84.9
MN	16.2 ^#^	8.12 ^#^	24.3 ^#^	66.8 ^#^	65.7	22.8	88.4	74.6
FN	14.6 ^#^	8.01 ^#^	22.6 ^#^	65.3 ^#^	74.7	23.1	97.8	76.3
SEM	0.507	0.349	0.543	1.311	1.935	1.038	2.044	1.034
Cr (additional)								
W (without)	20.9 ^a^	5.21 ^b^	26.2	80.8 ^a^	88.4 ^a^	16.8	105 ^a^	84.4 ^a^
P (picolinate)	20.2 ^a^	3.71 ^b^	23.9	84.7 ^a^	88.7 ^a^	17.2	106 ^a^	84.0 ^a^
N (nano)	15.4 ^b^	8.07 ^a^	23.4	66.0 ^b^	70.1 ^b^	23.0	93.1 ^b^	75.4 ^b^
p value	<0.001	<0.001	0.055	<0.001	<0.001	0.066	0.022	0.001
D (diet type)								
M	20.2 ^a^	6.24	26.4 ^a^	76.2	76.6 ^b^	19.0	95.7 ^b^	80.2
F	17.5 ^b^	5.09	22.6 ^b^	78.1	88.2 ^a^	18.9	107 ^a^	82.3
*p* value	<0.001	0.062	<0.001	0.362	0.002	0.975	0.007	0.335
Interaction Cr × D								
*p* value	0.508	0.404	0.235	0.430	0.855	0.902	0.850	0.972

* Details about the feeding period and how the experiment was performed were described in the Materials and Methods section. M, type of diet that includes a change from a high-fat diet to a low-fat diet; F, type of diet that includes staying on a high-fat diet for the entire duration of the experiment; C, rats fed a standard diet for laboratory rodents; W, rats fed a diet without supplementation with chromium; P, rats fed a diet with chromium picolinate; N rats fed a diet with chromium nanoparticles. ^a,b^ Mean values within a row with different superscript letters were significantly different (*p* < 0.05, two-way ANOVA followed by Duncan’s multiple range test). ^#^ Data significantly different from the control C group (*p* < 0.05, *t*-test).

**Table 7:** The activity of bacterial β-glucuronidase and β-xylosidase and their release rate in the cecum of rats (n = 10 per group) *.

	β-Glucuronidase				β-Xylosidase			
	Extracellular	Intracellular	Total	Release Rate	Extracellular	Intracellular	Total	Release Rate
Control C	32.2	17.1	49.3	65.0	4.85	6.39	11.2	43.4
2-way ANOVA:								
MW	40.9	19.6	60.5	67.2	5.05 ^a^	6.57	11.6	45.3
FW	47.3 ^#^	21.1	68.4 ^#^	68.7	2.67 ^b #^	5.23	7.90 ^#^	40.0
MP	39.8	20.1	60.0	66.3	3.49 ^b #^	6.34	9.83	37.0
FP	47.7 ^#^	23.5	66.2 ^#^	64.8	2.85 ^b #^	5.49	8.34 ^#^	35.3
MN	34.5	31.1 ^#^	65.6 ^#^	55.5	2.50 ^b #^	6.42	8.93	28.2 ^#^
FN	36.2	23.8	60.0	60.7	2.24 ^b #^	6.42	8.66	29.4
SEM	1.342	1.192	1.903	1.148	0.195	0.316	0.350	2.164
Cr (additional)								
W (without)	44.1 ^a^	20.3	64.4	67.9 ^a^	3.86	5.90	9.76	42.7
P (picolinate)	41.3 ^a^	21.8	63.1	65.5 ^a^	3.17	5.91	9.08	36.2
N (nano)	35.4 ^b^	27.5	62.8	58.1 ^b^	2.37	6.42	8.79	28.8
p value	0.045	0.071	0.944	0.004	0.002	0.802	0.536	0.073
D (diet type)								
M	38.4	23.6	62.0	63.0	3.68	6.44	10.1 ^a^	36.8
F	42.1	22.8	64.9	64.7	2.58	5.71	8.30 ^b^	34.9
*p* value	0.203	0.751	0.500	0.475	0.001	0.323	0.014	0.699
Interaction Cr × D								
*p* value	0.774	0.216	0.361	0.527	0.024	0.753	0.149	0.589

* Details about the feeding period and how the experiment was performed were described in the Materials and Methods section. M, type of diet that includes a change from a high-fat diet to a low-fat diet; F, type of diet that includes staying on a high-fat diet for the entire duration of the experiment; C, rats fed a standard diet for laboratory rodents; W, rats fed a diet without supplementation with chromium; P, rats fed a diet with chromium picolinate; N rats fed a diet with chromium nanoparticles. ^a,b^ Mean values within a row with different superscript letters were significantly different (*p* < 0.05, two-way ANOVA followed by Duncan’s multiple range test). ^#^ Data significantly different from the control C group (*p* < 0.05, *t*-test).

## Data Availability

Data supporting the reported results are available on request.

## Supplementary Materials

The following supporting information can be downloaded at: https://www.mdpi.com/article/10.3390/nu15143118/s1, Supplemental File S1: Body weight, feed intake, and NMR (nuclear magnetic resonance) parameters in rats fed experimental diets.
