# Supplementary material for: Effect of Chromium Nanoparticles and Switching from a High-Fat to a Low-Fat Diet on the Cecal Microenvironment in Obese Rats
Source: Nutrients. 2023 Jul 12;15(14):3118. doi: 10.3390/nu15143118 (PMC10384463; doi:10.3390/nu15143118)
Supplement: Supplementary file 1 [file nutrients-15-03118-s001.zip › nutrients-2427111-supplementary.pdf]

## Supplement S1

**Body weight, feed intake and NMR (nuclear magnetic resonance) parameters in rats fed experimental diets.**

The feeding period consisted of an initial 9 wk and an experimental 9 wk period. During the initial period, C rats were fed a standard low-fat diet (diet C), while the remaining groups (M, F, MP, FP, MN, FN) were subjected to an obesogenic high-fat diet (diet F). The dietary treatments used in the experimental period: Group C, control fed a C-diet; M, fed a C-diet; F, fed an F-diet; MP, fed a C-diet with Cr-Pic supplementation; FP, fed an F-diet with Cr-Pic; MN, fed a C-diet with Cr-NP; FN, fed a F-diet with Cr-NP. Both forms of Cr were added in a dose of 0.3 mg/kg body weight (BW). Two-way ANOVA showed that rats F excelled on the M ones with respect to the final BW, and they had a lower daily feed intake and a lean tissue mass (Table 1). A significant Cr×D interaction showed that the highest daily BW gain was attributed to the F and FP rats, while the lowest was attributed to the M, MP, and MN rats ($p < 0.05$). It should be stressed that the daily BW gain of group FN was significantly higher than in the MN rats but lower versus rats FP ($p < 0.05$). A Cr×D interaction was also noted for fat tissue mass, fat and lean tissue percentage (relative mass), and relative eWAT mass. The fat tissue mass was the highest in the F and FP rats ($p < 0.05$ vs. other experimental groups, including FN rats). When comparing the dietary counterparts fed the same Cr type (i.e., without Cr, with Cr picolinate, and Cr nanoparticles), only the MN and FN groups did not differ significantly between each other regarding the fat tissue mass and fat tissue percentage. The lean tissue percentage for the Cr×D interaction was higher in the FN group than in the FP and F groups ($p < 0.05$). Additionally, the FN group did not differ significantly compared with the MN and MP groups ($p > 0.05$). The Cr×D interaction showed the highest relative mass of the eWAT (epididymal white adipose tissue) in the F rats ($p < 0.05$ vs. all other groups except the FP). The MN and FN counterparts did not differ significantly.
